# New lupeol esters as active substances in the treatment of skin damage

**DOI:** 10.1371/journal.pone.0214216

**Published:** 2019-03-28

**Authors:** Magdalena Malinowska, Barbara Miroslaw, Elzbieta Sikora, Jan Ogonowski, Agnieszka M. Wojtkiewicz, Maciej Szaleniec, Monika Pasikowska-Piwko, Irena Eris

**Affiliations:** 1 Institute of Organic Chemistry and Technology, Cracow University of Technology, Cracow, Poland; 2 Department of Crystallography, Faculty of Chemistry, Maria Curie-Sklodowska University, Lublin, Poland; 3 Jerzy Haber Institute of Catalysis and Surface Chemistry, Polish Academy of Sciences, Cracow, Poland; 4 Dr Irena Eris Centre for Science and Research, Piaseczno, Poland; University of Minnesota Twin Cities, UNITED STATES

## Abstract

The purpose of the research was to obtain new derivatives of natural triterpene lupeol and to evaluate their potential as active substances in the treatment of skin damage. Four new lupeol esters (propionate, succinate, isonicotinate and acetylsalicylate) and lupeol acetate were obtained using an eco-friendly synthesis method. In the esterification process, the commonly used hazardous reagents in this type of synthesis were replaced by safe ones. This unconventional, eco-friendly, method is particularly important because the compounds obtained are potentially active substances in skin care formulations. Even trace amounts of hazardous reagents can have a toxic effect on damaged or irritated tissues. The molecular structure of the esters were confirmed by ^1^H NMR, ^13^C NMR and IR spectroscopy methods. Their crystal structures were determined using XRD method. To complete the analysis of their characteristics, physicochemical properties (melting point, lipophilicity, water solubility) and biological activity of the lupeol derivatives were studied. Results of an irritant potential test, carried out on *Reconstructed Human Epidermis* (RHE), confirmed that the synthesized lupeol derivatives are not cytotoxic and they stimulate a process of human cell proliferation. The safety of use for tested compounds was determined in a cell viability test (cytotoxicity detection kit based on the measurement of lactate dehydrogenase activity) for keratinocytes and fibroblasts. The results obtained showed that the modification of lupeol structure improve its bioavailability and activity. All of the esters penetrate the *stratum corneum* and the upper layers of the *dermis* better than the maternal lupeol. Lupeol isonicotinate, acetate and propionate were the most effective compounds in a stimulation of the human skin cell proliferation process. This combination resulted in an increase in the concentration of cells of more than 30% in comparison to control samples. The results indicate that the chemical modification of lupeol allows to obtain promising active substances for treatment of skin damage, including thermal, chemical and radiation burns.

## Introduction

Human skin, as the biggest organ in the human body, acts as a protective barrier. Among other, it prevents toxic substances from penetrating into deeper, more hydrophilic skin structures. Simultaneously, it allows drug molecules to diffuse through skin layers during the transdermal delivery [1]. The maintenance of skin integrity and function is especially important. Unfortunately, there are many factors that negatively affect the condition of the skin, causing its damage and dysfunctions. One of the reasons of skin barrier damage is the exposure to UV radiation. For example, UVB light causes many harmful effects including irritation, redness and burning of the skin. UVA is responsible for the damage of DNA and cell structure, skin aging and discoloration caused by free radicals. Other external conditions, including air pollution, electronic device radiation (blue ray) and the presence of controversial self-care product ingredients, may cause skin damage. Psoriasis, atopic dermatitis, skin allergies and inflammatory reactions are nowadays a problem which is affecting more and more people. Another cause of skin damage is burn injury, which can be caused by heat, radioactivity, electricity, friction or contact with chemicals [2]. What is more, cancer treatments using radiotherapy are a source of increasing numbers of skin damage. Some of cancer therapies create an extensive surface area of wounds that are difficult to heal.

Considering the importance of the barrier function of the skin, there is an urgent need to develop active ingredients with an intensive regenerative effect, which will simultaneously protect the damaged skin structure from external factors.

Triterpenes are promising agents for curing skin burns and accelerating the skin regeneration process. Their spectrum of biological activity is wide: antioxidant [3], anticancer [4,5], antibacterial [6,7], antivirus [8,9] and regulating melanin biosynthesis [10]. They are used in the treatment of various skin ailments [10–12]. One of the most popular triterpenes is lupeol, generally obtained by extraction processes from natural sources such as: birch bark, white cabbage, green pepper, olive oil, strawberries, mangoes or grapes [13]. Lupeol is already known as a compound stimulating skin cells proliferation and having influence on their migration, improving the damaged skin reconstruction. It modifies the refraction capacities of normal fibroblasts and increases isometric forces of fibroblasts from stretch marks. Moreover, no stress fibers are observed and the skin is stimulated to reconstruction under lupeol treatment [14]. The latest studies relating to the cosmetic or pharmaceutical application of lupeol-rich extract are focused on treating and preventing a connective tissue degeneration [15,16].

It is well known that the suitability for the active substances to be used in pharmaceutical or cosmetic applications is not only determined by its therapeutic activity, but also by their absorption, distribution, metabolism, excretion and toxicity (ADMET). In the case of topical application, the permeability of skin is a significant factor. The *stratum corneum* (the outermost layer of the skin) is highly lipophilic. It mostly consists of corneocytes and the intercellular spaces are filled with lipid compartments. Its character does not allow hydrophilic compounds to penetrate through it. Furthermore, the penetration of highly lipophilic components is also limited [1]. The presence of lipids in the *stratum corneum* is very significant when determining the affinity of highly lipophilic substances for the skin layer. The average lipids content of human *stratum corneum* is about 16%. Additionally, the main class of lipids in the *stratum corneum* are ceramides, cholesterol and fatty acids [17]. The structural similarity of lupeol and cholesterol (the main, representative compound of sterols group) could facilitate the triterpene compound to cross the skin barrier and enable the drug penetration through the *stratum corneum* [18]. As previously mentioned, many triterpenes exhibit significant biological activity, but some of their physicochemical properties can restrict their pharmaceutical and cosmetic use [12]. The need for effective treatments is increasing. Lupeol is known to have many benefits for damaged skin. However, it should be noted that despite the wide range of lupeol biological activity, due to high lipophilicity and poor solubility, its bioavailability is limited [19].

**Fig 1:**
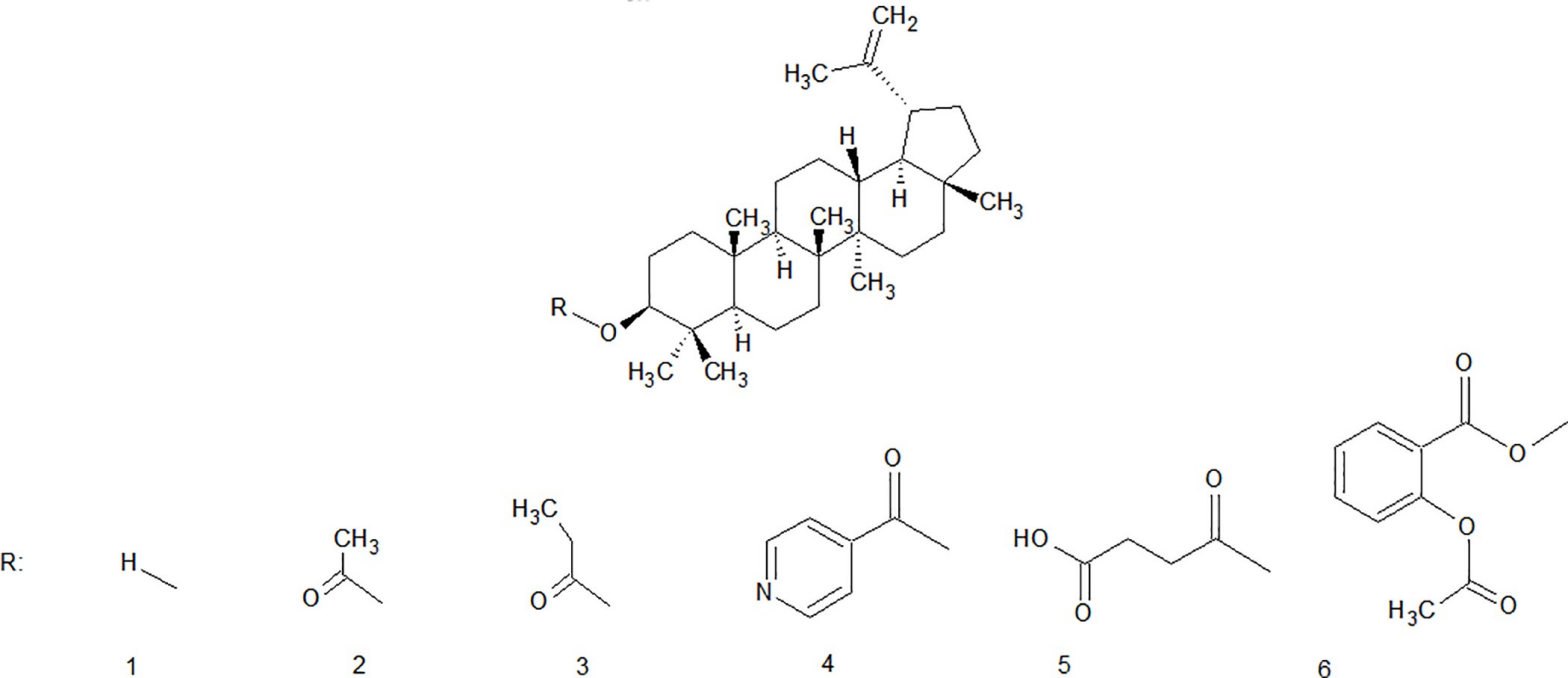
**The structures of lupeol (1) and synthesized esters (2-lupeol acetate, 3-lupeol propionate, 4-lupeol isonicotinate, 5-lupeol succinate, 6-lupeol acetylsalicylate)**.

In some cases, a modification of the active substances structure can increase their penetration through the skin. The purpose of our studies was to obtain new derivatives of lupeol and to evaluate their biological activity as potential agents dedicated for treatment of skin damage. Five lupeol esters (2–6) (Fig 1) were evaluated for their effectiveness in topical formulations. Four of the obtained compounds (3–6) were not described in scientific studies before. In order to compare the bioavailability of lupeol and its esters, their cytotoxicity, cell proliferation stimulation activity and the ability to penetrate the *epidermis* were tested.

## Materials and methods

### The synthesis and the physicochemical characteristic of lupeol esters

The lupeol derivatives were obtained by an esterification process. Appropriate carboxylic acid or carboxylic acid anhydrides were used as acylating agents to obtain lupeol esters: acetate (2), propionate (3), isonicotinate (4), succinate (5) and acetylsalicylate (6). In the first stage of the esterification process, lupeol (1 g; 2.3 mmol, Natchem) was dissolved in tetrahydrofurane (10 cm^3^, Avantor), then N-methylmorpholine (7.5 cm^3^, Sigma Aldrich) and a stoichiometric excess (2 eq.) of carboxylic acid or its anhydride (all from Sigma Aldrich) was added to the reaction mixture. Next, the mixture of N,N-dicyclohexylcarbodiimide and 4-dimethylaminopyridine (DCC-DMAP, both supplied by Sigma Aldrich) was applied as a catalyst (1.15 eq). The reactions were carried out in reflux for 4 hours.

The progress of the reactions was controlled using the Thin Layer Chromatography (TLC) method. Polygram Sil G/UV 254, Macherey Nagel TLC plates were used. The mixture of chloroform (Chempur) and ethyl acetate (Avantor) (9:1 v/v) was applied as an eluent. The TLC plates were sprayed with 50% water solution of phosphoric acid (Chempur) with addition of 10% isopropanol (Avantor) solution of vanillin (Sigma Aldrich) (10:1 v/v). Triterpenes became visible after heating the plate at 100°C for 2–3 minutes.

In each of the synthesis, the reaction mixtures were poured into 300 cm^3^ of 10% aqueous solution of hydrochloric acid (Avantor). The organic phase was neutralized by washing it with 20% aqueous solution of sodium bicarbonate (Avantor) for three times (50 cm^3^ each time). After that, the organic phase was dried with magnesium sulfate (Avantor) and concentrated to 1/3 of the original volume. The pure reaction products were precipitated from the solution as a white solids. After the solvent evaporation, the solids were dried to a constant mass, at room temperature and then crystallized from methanol and chloroform.

The melting points of the crystalline triterpenes were determined by Stuart SMP10 melting point apparatus. The UV-VIS spectra of the esters were measured using Macherey Nagel Nanocolor UV-VIS Spectrophotometer (in the wavelength range from 200 to 400 nm). The applied concentration of each triterpene amounted to 2 mg/cm^3^. Ethanol was used as a solvent and as a blank sample. The ^1^H NMR and ^13^C NMR analysis were recorded in CDCl_3_ using Mercury-VX Varian, 300 and 75 MHz, respectively. IR spectra were measured for chloroform solutions by Nicolet S10 Spectrometer. The 2 μL injections of the 0.1 mg/cm^3^ samples dissolved in acetonitrile were analysed by RP-HPLC-MS method, using Ascentis Express RP-Amide column (2.7 μm, 7.5 cm × 2.1 mm), in a gradient mode (80–98% acetonitrile/water/0.1% formic acid for 7 min followed by isocratic 98% of acetonitrile for next 20 min, flow rate 0.4 cm^3^/min, temperature 40°C). The MS detection (Agilent VL) was conducted in positive APCI ion mode with a mass range of 300–450 m/z, drying gas flow rate of 6 dm^3^/min, temperature 350°C, nebulizer pressure 60 psig, vaporizer temperature 500°C, capillary voltage 5000V (positive and negative) and corona current in the range of 5.0 μA.

Crystal data for 2, 3 and 5 were collected at 120 K on a SuperNova diffractometer equipped with the microfocus X-ray source and AtlasS2 detector, using the Cu Kα radiation (λ = 1.54184 Å). The CRYSALIS program system [20] was used for data collection, cell refinement and data reduction. The absorption corrections were applied by multi-scan method of Blessing [21]. Using Olex2 and ShelXS [22,23] the structures were solved using direct methods and refined with the ShelXL refinement package [23]. Due to very poor crystals of lupeol succinate (5), the disordered oxygen atoms were refined isotropically. The hydrogen atoms were introduced at calculated positions and refined riding on their carrier atoms. The crystals were enantiomerically pure. The absolute configuration of the chiral molecules was determined by using the Flack *x* [24] and Hooft *y* [25] parameters; however, they were of minor importance because the compounds were weak anomalous scatters. Crystal data and structure refinement for three lupeol esters: acetate, propionate and succinate are presented in Table 1. Crystal data for lupeol isonicotinate (4) and lupeol acetylsalicylate (6) were not obtained because of the non-crystalline form of the compounds.

**Table 1:** Lupeol esters physicochemical properties (MP–melting point, RT- retention time, RF—retardation factor).

No	Name	MP [ºC]	Purity	Reaction yield [%]	UV/VIS max [nm]	RT [min]	RF [–]
**1**	Lupeol	213–215	96.8	-	208.8	8.9	0.65
**2**	Lupeol acetate	216–219	95.1	85.4	207.8	9.0	0.72
**3**	Lupeol propionate	220–222	93.3	70.9	208.5	10.2	0.78
**4**	Lupeol isonicotinate	179–183	95.7	66.4	209.8	10.4	0.81
**5**	Lupeol succinate	221–223	96.2	69.2	208.3	8.9	0.68
**6**	Lupeol acetylsalicylate	226–229	92.2	53.0	209.3	9.4	0.77

### The evaluation of lipophilicity and potential therapeutic efficiency

Lipophilicity as one of the most important physicochemical property that influences skin barrier permeation should be always considered when new dermatological active ingredients are synthesized [26]. The lipophilic character of the tested triterpenes was characterized experimentally and theoretically. The experimental lipophilicity of the tested esters was determined through the calculation of chromatographic partition coefficients (RM), using reversed phase thin layer chromatography (RP-TLC) [27]. Assays were performed in duplicate on reverse-phase thin-layer aluminum sheets: RP-18F254S (Merck), 20 × 20 cm. The developing system was a mixture of 1,4-dioxane-acetate buffer (pH = 4.8), with concentrations of 1,4-dioxane in the range of 60–90% (v / v) with a graduation rate of 5%. Buffer pH was measured with the Seven Multi pH meter (Mettler Toledo). The chloroform solutions of the lupeol esters were applied to a TLC plate and developed at 23 ± 1˚C. After drying, the chromatograms were generated by spraying with 20% sulfuric acid in methanol and heating at 100˚C for 2–3 min. Retention factors (Rf) of the visible spots obtained in this process were calculated according to Pyka and Miszczyk [28].

The results were compared to the theoretical prediction methods such as ACD Labs Chemsketch 2012, v.14.01 [29], PubMed [30], Discovery Studio ver. 4.1 (BIOVIA) [31] databases and ChemAxon programme [32]. The PubMed database was not suitable to find values of two of the lupeol esters, propionate and acetylsalicylate, as these compounds were not described in scientific papers before. The theoretical water solubility (logSw) of the compounds obtained was used as a complementary parameter. LogSw was calculated according to Cheng and Merz predictive model which gives information about compound ADMET [33].

ADMET calculations were prepared using PreADMET 2004, v. 1.0 [34]. The calculations of toxicity of the described triterpenes were evaluated by Acute toxicity to Daphnia. Their solubility in buffer was calculated for pH 7.4 buffer system by SK atomic types (SKlogD distribution coefficient).

### The evaluation of cytotoxicity and proliferation activity

A Skin Irritation Test was prepared according to SOP (*Standard Operation Protocol*) of *In vitro* Skin Irritation Test (ECVAM): Human Skin Model, EpiDerm-200, Version: 7.0, 30^th^ Oct 2007. Reconstructed Human *Epidermis* (RHE) model represents place where a potential irritating substance acts and it shows the inflammation process which can appear after the exposure of the tissue to the irritating chemicals *in vivo* conditions. The method is based on cells viability assay and give information if tested compound shows irritating effect and if it can be classified into 2^nd^ category according to UN GHS (United Nations Globally Harmonized System) and EU CLP (Globally Harmonized System of Classification and Labelling of Chemicals) requirements [35].

Standard EpiDermFT kit (MatTek Corporation) consisted of 24 tissues. Costar Snapwell single well tissue culture plate inserts had the surface area of about 1.0 cm^2^ and the diameter of about 1.2 cm. Moreover inserts were equipped with pores (size 0.4 μm diameter). Before test, all tissues were visually inspected if there are any physical imperfections. The media used throughout the production process were checked for sterility. Furthermore, all cells were screened and were negative for HIV, hepatitis B and hepatitis C using PCR (all the tests made by MatTek). The culture medium was fed through a microporous membrane. The tissue contained 8–12 cell layers plus stratum corneum (basal, spinous, and granular layers) [35].

**Fig 2:**
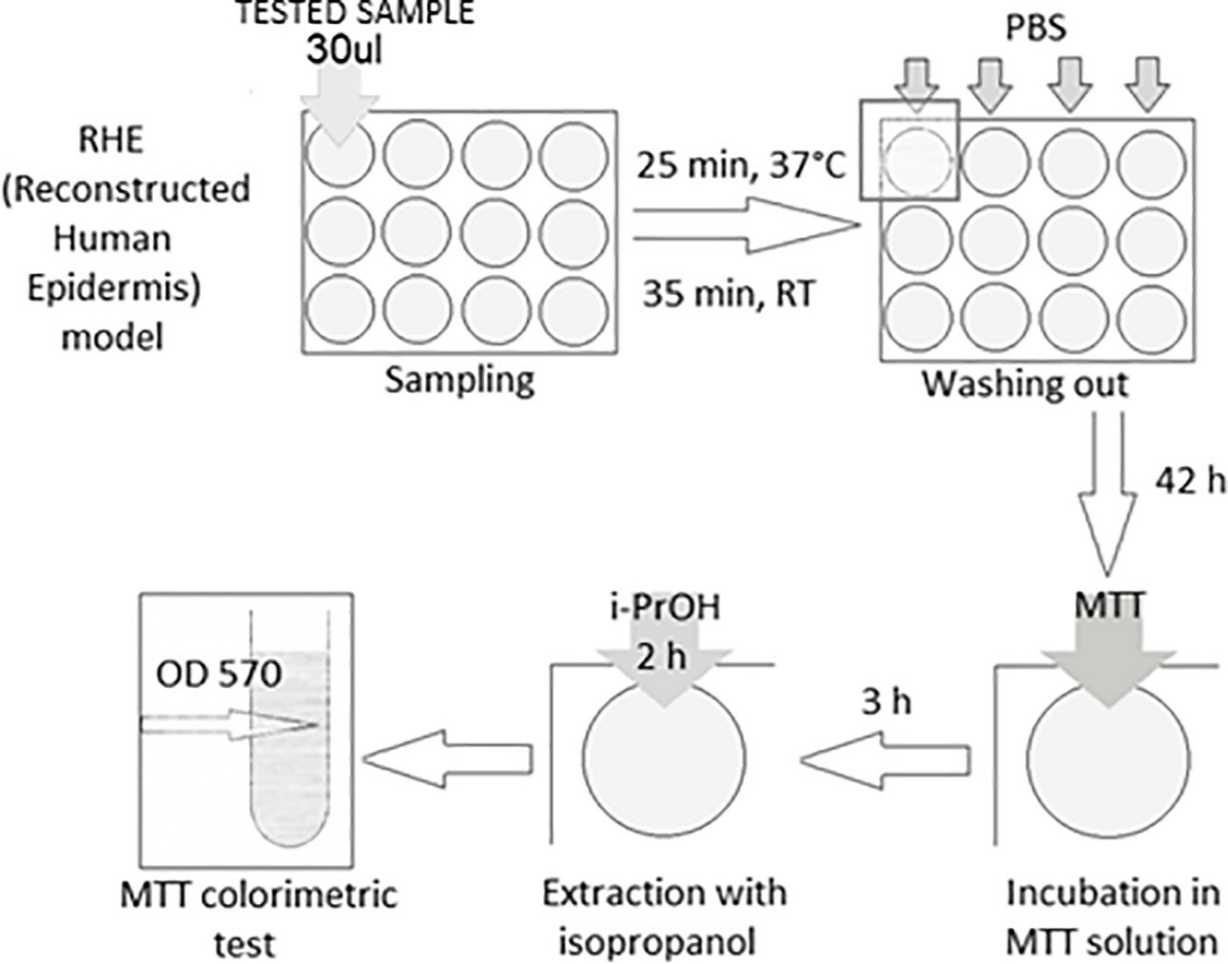
General scheme of the experiment with RHE (Reconstructed Human *Epidermis*) model and lupeol derivates (PBS–phosphate buffer, i-PrOH–isopropanol, RT–room temperature, MTT—3-(4,5-methylthiazol-2-yl)-2,5-diphenyltetrazolium bromide, OD–optical density).

*In vitro* skin irritation test was prepared according to the procedure described by Kandarova et al. [35]. On the first day, tissues were topically exposed to the oil solutions of tested triterpenes. White crystals of each compound were dissolved in caprylic and capric triglyceride (Crodamol GTCC, Croda). The esters concentration in the solutions was 0.5% (m/m). Three tissues were used for each tested substance, as well as for the positive and negative control. Positive control solution consisted of 5% aq. solution of SDS (Sodium Dodecyl Sulfate, Mattek), and a negative control solution consisted of Caprylic and Capric Triglycerides (Crodamol GTCC, Croda). Chemical exposure time was 60 min, during which, for 35 min, the tissues were kept in an incubator at 37°C (chamber Heraeus, Kendro). The test substances were then removed from the tissue surface by an extensive washing procedure using DPBS (phosphate-buffered saline, pH = 7.4, Mattek). The tissue inserts were blotted and transferred to fresh medium. A scheme of the procedure is shown in Fig 2.

After 24 hours of incubation, the medium was exchanged and the tissues were incubated for an additional 18 hours. After 42 hours of incubation time, the tissues were transferred into a yellow solution of MTT (3-(4,5-dimethylthiazol-2-yl)-2,5-diphenyltetrazolium bromide, Mattek) and incubated for a further 3 hours. The resultant purple-blue formazan salt, formed mainly by mitochondrial metabolism, was extracted after 2 hours with isopropanol, pure (Mattek). The optical density (OD570) of the extracted formazan solution was determined using a spectrophotometer (Biotek, PowerWave XS). Cells viability assay was calculated referring to the negative control tissues. A substance is classified as an irritant if the tissue viability relative to the negative control treated tissues is reduced below 50%.

The cytotoxicity assay was used to determine the toxicity of lupeol esters. Cell viability was assessed by the Cytotoxicity Detection Kit (Roche), a colorimetric assay based on the measurement of lactate dehydrogenase (LDH) activity released from the cytosol of damaged cells into the supernatant. The method is based on the reduction of MTS tetrazolium compound by viable cells to generate a colored product soluble in cell culture media. The assay was performed according to the manufacturer’s protocol. Cells were seeded in 96-well plates (fibroblasts) or in 12-well plates (keratinocytes). Keratinocytes isolated from the *epidermis* from 3 different donors were plated in KGM-Gold medium (Lonza) at a density of 5–20 x 10^3^ per cm^2^. Fibroblasts isolated from the *dermis* from 3 different donors were plated in DMEM medium supplemented with 10% fetal bovine serum at a density of 5–20 x 10^3^ per cm^2^. After 24 hours, the medium was changed to fresh with the addition of lupeol esters at concentrations of 100μM in dimethyl sulfoxide (DMSO, Sigma Aldrich). Cells were than incubated with test substances or vehicle control for 48 hours (37°C, 5% CO_2_, 95% humidity). Maximum LDH release was induced with 1% (v/v) Triton X100 in assay medium. The absorbance of the samples was measured at 490 nm with the reference wavelength at 620 nm using the VersaMax ELISA Microplate Reader (Molecular Devices). The experiments were run in triplicate. The cytotoxicity of the tested compounds was determined by the estimation of cells viability of incubated samples compared to the control.

## Results and discussion

### The synthesis and the physicochemical characteristic of lupeol esters

Four new lupeol derivatives (propionate, succinate, isonicotinate and acetylsalicylate) and lupeol acetate were obtained by lupeol esterification using various acids or acid anhydrides.

As mentioned previously, compounds 3–6 (Fig 1) are novel structures. Compound 2 (lupeol acetate) has been described in the literature as biologically active lupeol derivative. It shows similar activity as lupeol but it exhibits better bioavailability. Lupeol acetate significantly decreases rheumatoidal arthritis symptoms by inhibition of inflammatory cytokines expression [19]. This anti-inflammatory activity is very significant during skin regeneration process. Chemical modification of lupeol can alter not only biological activity of the obtained derivative but can also improve its bioavailability and effectiveness. The general procedure of lupeol esterification described by Vasnev *et al*. [36] requires the application of catalysts and solvent with relevant high cytotoxicity. The conventional reaction is carried out in dichloromethane and pyridine. DMAP (4-dimethylaminopyridine) plays, in the reaction, the role of a catalyst. Considering the application of the synthetized compounds as the active ingredients in skin care products, these hazardous substances were replaced by another less harmful and safer ones.

Table 1 presents the values of the melting points, purities, reaction yields and maximum absorbance wave lengths of the tested compounds. The esterification yields ranged from 64.8 to 87.9% which is satisfactory in comparison to similar synthesis of lupeol derivatives [37,38]. All of the obtained esters had form of white solids. The purity of the obtained compounds was relatively high (92.9–96.2%), especially as the substrate, natural lupeol (96.8%) was most probably contaminated with other triterpenes and secondary birch metabolites. An extraction of the natural lupeol from plant material may result in the presence of other lipophilic triterpene compound like 3-*epi*-lupeol, α-amyrin or β-amyrin [39]. Melting points of the obtained esters were higher than the temperature of the alcohol substrate. The narrow range of the melting points values confirmed a high purity of the synthesised substances.

The molecular structure of the compounds obtained were confirmed using spectroscopic methods (1HNMR, 13CNMR, IR) and by MS-APCI and CHN methods (see: S1 File). MS-APCI analysis shows that there are insignificant impurities present in lupeol esters which are not visible at lupeol chromatogram. Intense ion mass signals of 409.3 ([M+H-H_2_O]^+^ of lupeol) and 423.3 m/z, which are also present in lupeol standard (analytical standard, Sigma Aldrich) are described by Khan *et al*. as characteristic for lupeol triterpene [40].

**Fig 3:**
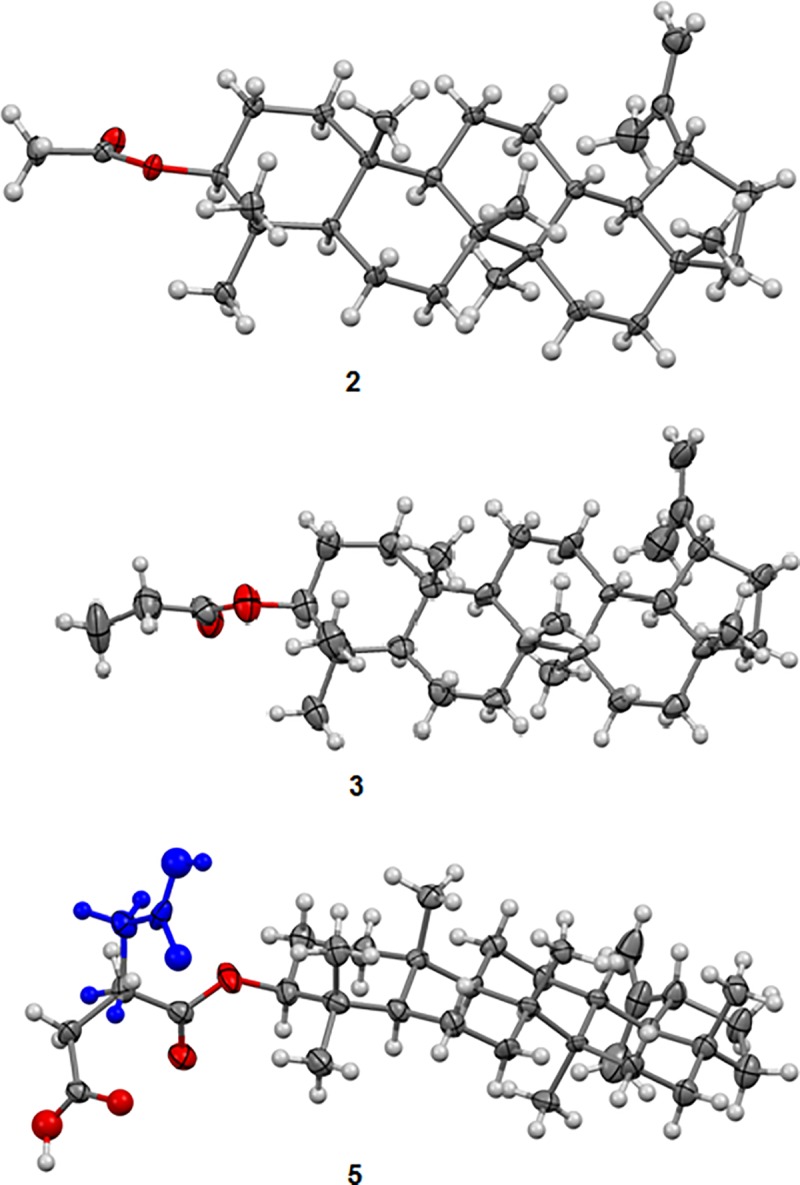
Molecular structures of 2 (lupeol acetate), 3 (lupeol propionate) and 5 (lupeol succinate) with shown two positions of the disordered ester group.

The X-ray crystal structure analysis of esters 2, 3 and 5 gave an unambiguous confirmation of the successful syntheses of new enantiopure esters of lupeol. The R–C (= O)–O–ester parts of the molecules are perpendicular to the mean plane of the triterpenoid fragment in (2) and (3), whereas in 5 the alkyl chain has a bent conformation with the carboxylic group being disordered in the crystal over two positions (Fig 3).

The positional disorder observed in compound 5 (lupeol succinate) is probably associated with the necessity to adjust the molecular conformation to the packing during crystallization of only one enantiomer. In all of the analysed crystals a “head to head” packing is observed as in the crystal of lupeol [1]. However, the introducing of the less hydrophilic ester groups instead of hydroxyl one, causes that the molecules interact mainly through weak C–H^…^O contacts. This change in intermolecular interactions explains a poor quality of ester crystals and simultaneously decreases their solubility in polar solvents. Only in lupeol succinate (5) the stronger intermolecular interactions are observed. The molecules form dimers through O–H^…^O hydrogen bonds between the disordered carboxylic groups. These results correlate well with the lipophilicity determination data, which show that lupeol succinate (5) is the less hydrophobic in the studied group of esters, whereas acetate (2) and propionate (3) are more lipophilic than lupeol (1).

The obtained crystal data and structure refinement for lupeol acetate (2), lupeol propionate (3) and lupeol succinate (5) are presented in Table 2.

**Table 2:** Crystal data and structure refinement for 2- lupeol acetate, 3- lupeol propionate and 5-lupeol succinate.

ID	2	3	5
Empirical formula	C_32_H_52_O_2_	C_33_H_54_O_2_	C_34_H_54_O_4_
Crystal system	orthorhombic	monoclinic	monoclinic
Space group	*P*2_1_2_1_2_1_	*C*2	*C*2
*a*/Å	8.0837(2)	14.341(2)	14.269(2)
*b*/Å	21.6854(5)	6.5377(7)	6.6401(8)
*c*/Å	47.385(1)	30.880(3)	31.787(5)
*β*/°	90	96.70(1)	96.31(1)
Volume/Å^3^	8306.5(3)	2875.4(5)	2993.6(7)
*Z*, *Z’*	12, 3	4, 1	4, 1
*ρ*_calc_g/cm^3^	1.124	1.115	1.169
*μ*/mm^-1^	0.508	0.502	0.576
*F*(000)	3120.0	1072.0	1160.0
Crystal size/mm^3^	0.4 × 0.08 × 0.05	0.3 × 0.15 × 0.05	0.3 × 0.3 × 0.05
Reflections collected	58157	20119	9772
Independent reflections	15034 [*R*_int_ = 0.0982, *R*_sigma_ = 0.0804]	5193 [*R*_int_ = 0.1028, *R*_sigma_ = 0.0751]	4597 [*R*_int_ = 0.0749, *R*_sigma_ = 0.0982]
Data/restraints/parameters	15034/0/967	5193/1/324	4597/1/366
Goodness-of-fit on *F*^2^	1.016	1.080	1.047
Final *R* indexes [*I*> = 2*σ* (*I*)]	*R*_1_ = 0.0564, w*R*_2_ = 0.1382	*R*_1_ = 0.0956, w*R*_2_ = 0.2182	*R*_1_ = 0.0938, w*R*_2_ = 0.2500
Final *R* indexes [all data]	*R*_1_ = 0.0685, w*R*_2_ = 0.1500	*R*_1_ = 0.0876, w*R*_2_ = 0.2385	*R*_1_ = 0.1129, w*R*_2_ = 0.2720
Largest diff. peak/hole / e Å^-3^	0.36/-0.26	0.52/-0.34	0.61/-0.44
Flack *x* parameter	0.1(2)	0.1(5)	0.2(5)
Hooft *y* parameter	0.1(2)	0.6(3)	0.1(4)
CCDC No.	1487997	1487998	1487999

### The evaluation of lipophilicity and potential therapeutic efficiency

RP-HPLC-MS method not only determined the purity of the triterpenes and confirmed the preservation of the lupeol core after chemical processing, but also revealed differences in esters hydrophobicity (Rf and RT values). Propionate and acetylsalicylate lupeol derivatives and to a lesser extent the lupeol acetate exhibit a higher hydrophobicity than the parental lupeol. Such a tendency was confirmed by the logP values (Table 3).

**Table 3:** Lupeol esters lipophilic properties (RM_0_, ACD/logP, XlogP3, AlogP and logSw), RT- retention time, Rf—Retardation factor.

No	Name	logP (RM_0_) RP-TLC method	ACD/logP	XlogP3 PubMed	AlogP	ACD/logD pH 5.5 / pH 7.4	Chemaxon logD pH 7.4	logSw
1	Lupeol	7.67+0.07	10.98+/- 0.38	9.9	7.403	9.41/ 9.41	7.45	-8.757
2	Lupeol acetate	8.12+0.03	11.87+/- 0.40	10.4	7.782	10.92/ 10.92	7.889	-9.565
3	Lupeol propionate	8.23+0.09	12.41+/- 0.40	-	8.449	-	8.59	-9.994
4	Lupeol isonicotinate	7.98+0.04	12,58+/- 0,41	-	7,627	-	8.73	-9.998
5	Lupeol succinate	8.34+0.08	11.49+/- 0.53	10.3	7.627	8.99/ 7.19	4.72	-8.479
6	Lupeol acetylsalicylate	9.76+0.08	13.17+/- 0.57	-	9.214	-	9.55	-10.005

Data in Table 3 show the lupeol esters lipophilicity, expressed as a logarithm of octanol–water partition coefficient (logP_OW_) determined by different methods: experimental (RP-TLC method) [27] and theoretical (ACD/logP [29], XlogP3 PubMed [30], AlogP and Chemaxon logP [32]). The values of logD were theoretically determined with Chemaxon and ACD Chemsketch [29].The values of logSw have been also theoretically evaluated [33]. The example relation between pH and logP as well as logD values are presented in S1 File. The calculations were prepared using Calculator Plugins of Chemicalize.

The data shown in the Table 3 indicate that in all cases the lipophilicity of the obtained lupeol esters is higher than the starting triterpene alcohol. The lowest values of octanol / water partition coefficient logarithm were obtained for lupeol succinate (from 7.623 to 11.49, according to applied method) and the most lipophilic compound was lupeol acetylsalicylate (logP values ranged from 9.214 to 13.17). Experimental values comply with the theoretical ones. Simultaneously, lupeol exhibited the lowest logP values (from 7.403 to 10.980) which shows that its structure modification influences its compound character.

Potential therapeutic agents are evaluated at early stages of drug development based on computational modeling, high throughput screening and cell-based assays that predict their pharmacologic activity. Predicting the compound absorption, distribution, metabolism, excretion and toxicity (ADMET) is much more complicated. The rule of five which is widely used to predict these processes for transdermal way of drugs application [41]. The studied triterpenes comply the three requirements of the Rule of Five: molecular weight MW (< 500), number of H-bond donors NHD (< 5) and number of H-bond acceptors NHA (< 10). The predicted values of octanol partition coefficient logarithm logP exceed the expected value logP < 5 (Table 2). All of the tested compounds are insoluble in water (logSw between -8.5 and -10.0) but highly soluble in organic solvents. The esterification of lupeol hydroxyl group increased the logP parameter. The permeation tests of lupeol, however, confirm that lupeol can absorb into skin structures [14,42]. The *stratum corneum* permeation tests show that the permeability for lipophilic compounds is not homogenous and generally decreases rapidly with depth. It has been diagnosed by Raman spectroscopy that the lipophilic compounds, like terpenes, act within *stratum corneum*. The absorption profile of terpene beta-carotene in the near IR region extends to about 800 nm [43]. The logP restriction which is described by the Rule of Five concerns molecules dedicated to the deeper skin layers including the *dermis* layer or drugs which are supposed to permeate into the bloodstream [40]. Compounds with high lipophilicity will not absorb into *dermis* but will act within *epidermis* layer. Therefore, high lipophilic character is crucial for biologically active substances that should act within the *stratum corneum* structures [1] and the studied triterpene compounds may penetrate the upper skin layers. Notably, the esterification of lupeol hydroxyl group causes the increase of logP wherein it is dependent on acyl group structure. Taking into consideration the fact that the *stratum corneum* is a highly lipophilic medium, triterpene compounds are suitable active substances that acts in upper skin layers. The values of logD for lupeol isonicotinate (4) and lupeol succinate (5) are dependent on pH values as shown for theoretical estimations (see S1 File). The calculations of these values can be based on the consideration of microscopic dissociation constants (microconstants), the partition coefficients of the microspecies for the compound and the counterion concentration [44]. However, our calculations suggests, that only in case of lupeol succinate (5) the slightly acidic pH of skin (5.0–5.5) will have significant influence on its hydrophobicity (i.e. increased value of logD due to partial protonation of free carboxyl group of succinate).

ADMET calculations were prepared using PreADMET 2004, v. 1.0. Numerous *in vitro* methods have been used in the drug selection process for assessing the transdermal absorption of lupeol triterpene and the obtained esters. Among others, the in silico skin permeability model could predict and identify potential drugs for transdermal delivery. The calculations of toxicity of the described triterpenes were evaluated by Acute toxicity to *Daphnia*. Tested compounds, as potential active ingredients in cosmetic and pharmaceutical industry, offer a wide range of benefits, but they also have a lot of limitations, such as potential environmental toxicity. When we consider new structures, which have not been present in natural environment before, it is important to ensure that level of their toxicity should be below the levels presenting an unacceptable risk to humans. Computational modeling approach such as Quantitative Structure—Activity Relationships (QSARs) are widely used to evaluate this property and *Daphnia* is a basic model considered for these calculations. The prime aim of the calculations is to generate models for accurate and reliable predictions of unknown compounds properties. Models are based on the experimental acute toxicity data of various compounds against *Daphnia*. After collection of the experimental data, they were carefully screened for particular endpoints and same exposure time in order to get reliable predictions from the standardized data [45].

Moreover, the solubility in phosphoric buffer (PBS) pH 7.4 was calculated for all of the compounds. The SKlog*D* values were calculated from the computed log*P* and p*K*a, according to the equation log*D* = log*P* − log (1+10p*H*−p*K*a) and pH = 7.4 [32] (Table 4).

**Table 4:** The selected ADMET calculations for lupeol (1) and its derivatives (2-lupeol acetate, 3-lupeol propionate, 4-lupeol isonicotinate, 5-lupeol succinate, 6-lupeol acetylsalicylate).

ID	1	2	3	4	5	6
**PBS (pH = 7.4) solubility [mg/dm^3^]**	1.95	2.00	0.82	0.17	24.38	0.03
**Water solubility [mg/dm^3^] x10^-3^**	1.42	0.67	0.18	0.32	1.63	0.01
**SKlogD value**	7.48	7.78	8.42	6.43	6.43	9.16
**Toxicity (48hrs, EC50 in mg/dm^3^) x 10^−3^**	3.05	1.70	1.33	1.06	1.55	0.29

Extremely low values of water solubility confirm lipophilic character of the tested triterpenes. On the other hand, higher buffer solubility (especially 24.38 mg/dm^3^ for lupeol succinate) provides the possibility of the application of this ester as aqueous phase ingredient in emulsion formulation. Low toxicity of the obtained lupeol esters leads to the conclusion that they are potentially safe components for cosmetic or pharmaceutical formulations for topical applications. All of the tested compounds exhibit excellent ability of binding to plasma protein. The plasma protein binding in all the cases was 100% and the values were calculated according to human albumin. The ability values confirm the therapeutic efficiency of the tested triterpene compounds.

### The evaluation of cytotoxicity and proliferation activity

The test of the compound irritancy was performed for three fragments of RHE model for each compound to obtain an estimation of its cytotoxic activity. Cells viability assay was calculated from mean optical density (OD570) value in colorimetric MTT test. Table 5 presents the cells viability assay for the tested triterpenes as well as for the positive and negative control. Repeatability of the obtained results for the positive control referring to the negative control was no higher than 20%. For the analysis in triplicates of the tested solutions, the repeatability was determined with standard deviation values.

**Table 5:** Viability assay of the cells *in vivo* conditions after exposure to tested triterpenes 1–6.

Method	Tested cells	Viability [%] / SD							
		1	2	3	4	5	6	NC	PC
Viability	*Epiderm*	89.2 /6.0	131.2/ 19.6	133/ 17.6	134.1/ 10.2	120.0/ 12.1	120.9/ 7.5	100.0/ 10.2	28.1/ 10.2
LDH	keratinocytes	91.6/ 0.4	98.1/ 2.5	94.7/ 3.1	101/ 0.5	93.2/ 4.0	91.5/ 2.1	101.0/ 2.3	-
	fibroblasts	100.0/ 0.5	100.9/ 0.4	101.1/ 0.2	101/ 0.69	102.0/ 0.6	101/ 0.7	100.6/ 0.7	-

(NC-negative control, PC–positive control), LDH cytotoxicity assay (SD-standard deviation, n = 3)

The data shown in Table confirm that all of the synthesized compounds have no irritating properties. Positive control (PC, 28.1% cells viability) used in cell viability assay caused cells destruction process. Negative control (NC—100% cells viability) was a reference sample which had no effect on cell destruction as well as their proliferation. The cells viability observed for lupeol was slightly lower than for NC. Whereas the proliferation activity for the esters reached over 30% of the initial cell concentration. The most effective in causing cell proliferation were compounds 2–4. (Table 5). Cytotoxicity LDH assay proves safety of use for all tested compounds. There were no toxic properties for human keratinocytes and fibroblasts observed for lupeol esters solutions.

## Conclusion

The chemical modification of lupeol is an efficient way to obtain new compounds with desirable properties. The esterification of lupeol’s hydroxyl group resulted in the increased lipophilicity of the obtained esters with respect to the initial compound, enabling the compounds to precisely target the hydrophobic skin sub-structures. None of the studied triterpenes showed cytotoxic activity. The chemical modification also changed the biological activity of the alcohol, giving the new compounds better skin recovery properties, stimulating human skin cells proliferation. Lupeol isonicotinate, acetate and propionate (2–4) were the most effective compounds in stimulating human skin cell proliferation resulting in more than 30% increase of cell concentration in comparison to control samples. The lupeol esters presented here show promising activity in stimulating skin repairing processes and can be applied as active substances in formulations for topical application especially in treatment of skin burns.

## Supporting information

S1 File Physicochemical properties of the obtained compounds: physical form, melting point, reaction yield, compound purity, absorption maximum, data for IR and NMR spectra interpretation, data for elementary analysis and MS-APCI analysis, logP and logD values at various pH. (DOCX)
