# Supplementary material for: New lupeol esters as active substances in the treatment of skin damage
Source: PLoS One. 2019 Mar 28;14(3):e0214216. doi: 10.1371/journal.pone.0214216 (PMC6438679; doi:10.1371/journal.pone.0214216)
Supplement: S1 File — (DOCX) [file pone.0214216.s001.docx]

**Supporting Information**

Full experimental data for lupeol and its semisynthetic esters structure analysis.

Lupeol (1) ^1^H NMR (CDCl_3_) δ [ppm]: δ4.61(dd, 2H, H-5) δ4.46(m, 1H, H-6) δ2.36(m, 1H H-2) δ2.04(s, 3H, H-23) δ1.88(m, 1H, H-9) δ1.66(m, 6H, H-27,28) δ1.58(m, 2H, H-3) δ1.50(m, 1H, H-11) δ1.45(m, 1H, H-17) δ1.38(dd, 4H, H-10,14) δ1.31(dd, 4H, H-8,13) δ1.15(m, 4H, H-18,16) δ1.02(s, 3H, H-24) δ0.97(d, 1H, H-21) δ0.93(s, 3H, H-26) δ0.84(m, 8H, H-22,30,31) δ0.79(d, 4H, H-19,20) δ0.06(s, 1H, H-29). ^13^C NMR (CDCl_3_) δ [ppm]: 150.91(C-25) 109.31(C-30) 78.96(C-6) 55.29(C-21) 50.42(C-31) 48.29(C-4) 47.96(C-5) 42.98(C-1) 42.81(C-7) 40.81(C-12) 39.99(C-15) 38.84(C-17) 38.70(C-11) 38.04(C-9) 37.15(C-2) 35.57(C-3) 34.27(C-20) 29.84(C-19) 27.98(C-10) 27.43(C-13) 27.40(C-14) 25.13(C-8) 20.92(C-16) 19.30(C-18) 18.31(C-7) 17.99(C-28) 16.11(C-22) 15.97(C-26) 15.36(C-23) 14.54(C-24). IR max (cm-1): 3310.74, 3066.94, 2943.03, 2898.01, 2871.65, 1638.03, 1466.61, 1451.95, 1387.01, 1379.01, 1367.10, 1347.77, 1334.61, 1305.35, 1297.73, 1278.32, 1255.05, 1241.65, 1189.20, 1139. 78, 1106.30, 1082.77, 1068.61, 1042.55, 1036.22, 1013.99, 992.55, 982.77, 972.30, 944.17, 916.86, 878.52, 860.83, 820.59, 806.13, 745.94, 764.21, 731.21 688.10, 659.75. Calculated for C30H50O (426.72): C, 84.44%; H, 11.81%; found: C, 84.39%; H, 12.29%. MS-APCI: m/z = 423.3 [M + H]+ (100%), calc 423.3


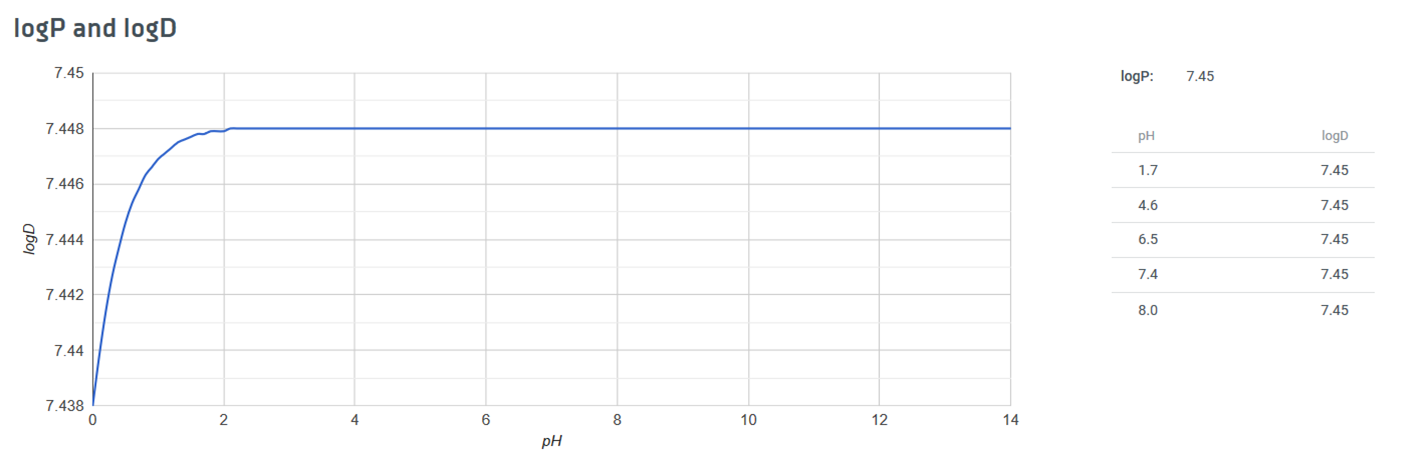


**Lupeol acetate (2) ^1^H NMR** (CDCl_3_) δ [ppm]: δ4.60 (m, 2H, H-5), δ3.18 (dd, 1H, H-6), δ2.37 (td, 1H, H-2), δ1.91 (m, 2H, H-3), δ1.70 (d, 1H, H-9), δ1.67 (d, 3H, H-23) δ1.64 (m, 2H, H-14), δ1.59 (m, 2H, H-10), δ1.51 (m, 1H, H-13), δ1.43 (dd, 1H, H-8), δ1.37 (m, 6H, H-27,28) δ1.25 (m, 4H, H-16,18), δ1.10 (m, 4H, H-19,20), δ1.02 (s, 3H, H-24), δ0.98 (m, 1H, H-17), δ0.96 (s, 3H, H-26), δ0.94 (s, 3H, H-22), δ0.87 (dd, 1H, H-11), δ0.82 (s, 3H, H-33), δ0.77 (d, 5H, H-30,31), δ0.67 (d, 1H, H-21). **^13^C NMR** (CDCl_3_) δ [ppm]: 170.95(C-32), 150.90(C-25), 109.33(C-30), 80.94(C-6), 55.36(C-21), 50.32(C-31), 48.27(C-4), 47.98(C-5), 42.97(C-1), 42.80(C-7), 40.83(C-12), 39.98(C-15), 38.37(C-17), 38.02(C-11), 37.77(C-9), 37.06(C-2), 35.55(C-3), 34.19(C-20), 29.82(C-19), 29.68(C-10), 27.93(C-13), 27.42(C-14), 25.08(C-8), 23.69(C-33), 20.93(C-16), 19.27(C-18), 18.19(C-27), 17.98(C-28), 16.48(C-22), 16.16(C-26), 15.96(C-23), 14.49(C-24). **IR max** (cm-1): 3073.64, 2939.48, 2871.85, 2855.57, 1731.57, 1639.59, 1451.85, 1365.73, 1318.25, 1244.46, 1199.12, 1176.43, 1147.02, 1102.05, 1015.17, 979.00, 944.95, 901.33, 874.26, 801, 36, 745.00, 703.73, 658.87. **Calculated for** C32H52O2 (468.75): C, 81.99%; H, 11.18%; found: C, 79.96%; H, 11.51%. **MS-APCI:** m/z = 423.3 [M + H]+ (100%), calc 423.3


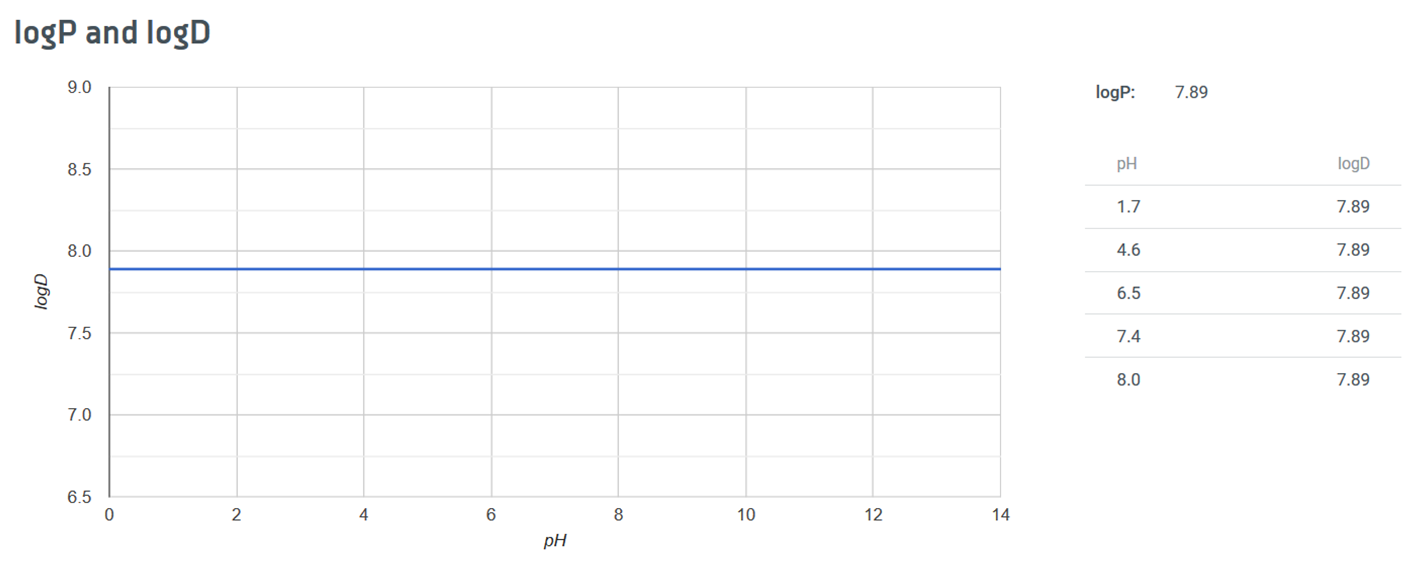


**Lupeol propionate (3)** **^1^H NMR** (CDCl_3_) δ [ppm]: δ4.68 (d, 2H, H-5), δ4.56 (dd, 1H, H-6), δ4.49 (m, 1H, H-2), δ4.22 (m, 1H, H-9), δ3.71 (m, 1H, H-3), δ3.65 (dd, 5H, H-33,35) δ2.65 (m, 4H, H-13,14), δ2.36 (ddd, 2H, H-10), δ1.91 (m, 2H, H-8), δ1.68 (d, 3H, H-23), δ1.63 (m, 4H, H-16,18) δ1.56 (s, 1H, H-11), δ1.49 (s, 1H, H-17), δ1.45 (d, 1H, H-21), δ1.39 (m, 6H, H-27,28), δ1.29 (m, 4H, H-19,20), δ1.02 (s, 3H, H-24), δ0.97 (s, 1H, H-21), δ0.93 (s, 3H, H-26), δ0.83 (s, 5H, H-30,31), δ0.78 (s, 3H, H-22). **^13^C NMR** (CDCl_3_) δ [ppm]: 174.25(C-32), 150.90(C-25), 109.33(C-30), 80.62(C-6), 55.35(C-21), 50.31(C-31), 48.26(C-4), 47.98(C-5), 42.97(C-1), 42.81(C-7), 40.82(C-12), 39.97(C-15), 38.35(C-17), 38.02(C-11), 37.85(C-9), 37.06(C-2), 35.55(C-3), 34.19(C-20), 29.81(C-19), 28.05(C-10), 27.93(C-13), 27.41(C-14), 25.07(C-8), 23.71(C-33), 20.92(C-16), 19.27(C-18), 18.18(C-27), 17.98(C-28), 16.52(C-22), 16.15(C-26), 15.96(C-23), 14.50(C-24), 9.33(C-35). **IR max** (cm^-1^): 3070.12, 2936.41, 2892.53, 2865.93, 2846.74, 1730.09, 1640.67, 1484.29, 1454.77, 1423.24, 1389.40, 1380.63, 1363.36, 1344.40, 1337.14, 1260.15, 1217.01, 1189.36, 1147.33, 1132.44, 1105.42, 1082.43, 1039.76, 1016.61, 975.37, 967.11, 942.38, 926.17, 910.24, 881.22, 803.48, 771.91, 739.92. **Calculated for** C33H54O2 (482,78): C, 82.10%; H, 11.27%; found: C, 81.78%; H, 11.62%. **MS-APCI**: m/z = 409.3 [M + H]+ (100%), calc. 409.4


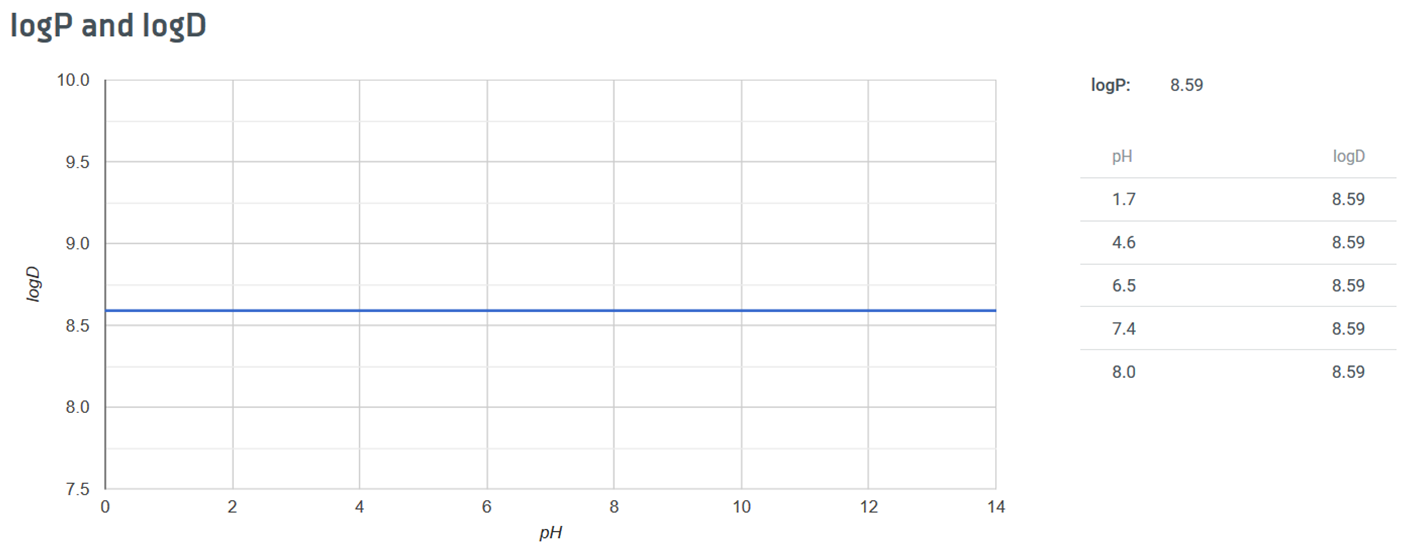


**Lupeol isonicotinate (4)** **^1^H NMR** (CDCl3) δ [ppm]: 9.00 (d, 2H, H-36,38), 8.37 (d, 2H, H-35,37), 4.81 (m, 1H, H-6), 4.57 (m, 2H, H-5), 2.37 (3td, 1H, H-2), 1.93 (m, 2H, H-3) 1.78 (s, 3H, H-23), 1.65 (m, 6H, H-27,28) 1.50 (d, 1H, H-9), 1.46 (d, 1H, H-11), 1.40 (m, 4H, H-10,14), 1.32(m, 4H, H-8,13), 1.16(m, 4H, H-16,18), 1.04 (s, 3H, H-24), 1.00 (s, 3H, H-26), 0.91 (t, 8H, H-22,30,31) 0.86 (d, 1H, H-21) 0.76 (m, 4H, H-19,20) 0.63 (m, 1H, H-17) **^13^C NMR** (CDCl3) δ [ppm]: 161.83(C-32) 150.90(C-25) 144.85(C-36) 143.59(C-38) 126.02(C-35) 109.35(C-30) 85.07(C-37) 78.93(C-6) 55.31(C-21) 50.36(C-31) 48.24(C-4) 47.95(C-5) 42.96(C-1) 42.81(C-7) 40.81(C-12) 39.95(C-15) 38.83(C-17) 38.68(C-11) 38.21(C-34) 37.98(C-9) 37.08(C-2) 35.51(C-3) 34.11(C-20) 29.80(C-19) 28.18(C-10) 27.98(C-13) 27.41(C-14) 25.03(C-8) 20.96(C-16) 19.28(C-18) 18.15(C-7) 17.97(C-28) 16.15(C-22) 15.96(C-26) 15.38(C-23) 14.50(C-24). **IR max** (cm^-1^): 3070.12, 2936.41, 2892.53, 2865.93, 2846.74, 1730.09, 1640.67, 1484.29, 1454.77, 1423.24, 1389.40, 1380.63, 1363.36, 1344.40, 1337.14, 1260.15, 1217.01, 1189.36, 1147.33, 1132.44, 1105.42, 1082.43, 1039.76, 1016.61, 975.37, 967.11, 942.38, 926.17, 910.24, 881.22, 803.48, 771.91, 739.92. **Calculated for** C36H53NO2 (531.81): C, 81.30%; H, 10.04%; found: C, 80.74%; H, 10.12%. **MS-APCI:** m/z = 423.3 [M + H]+ (100%), calc 423.3


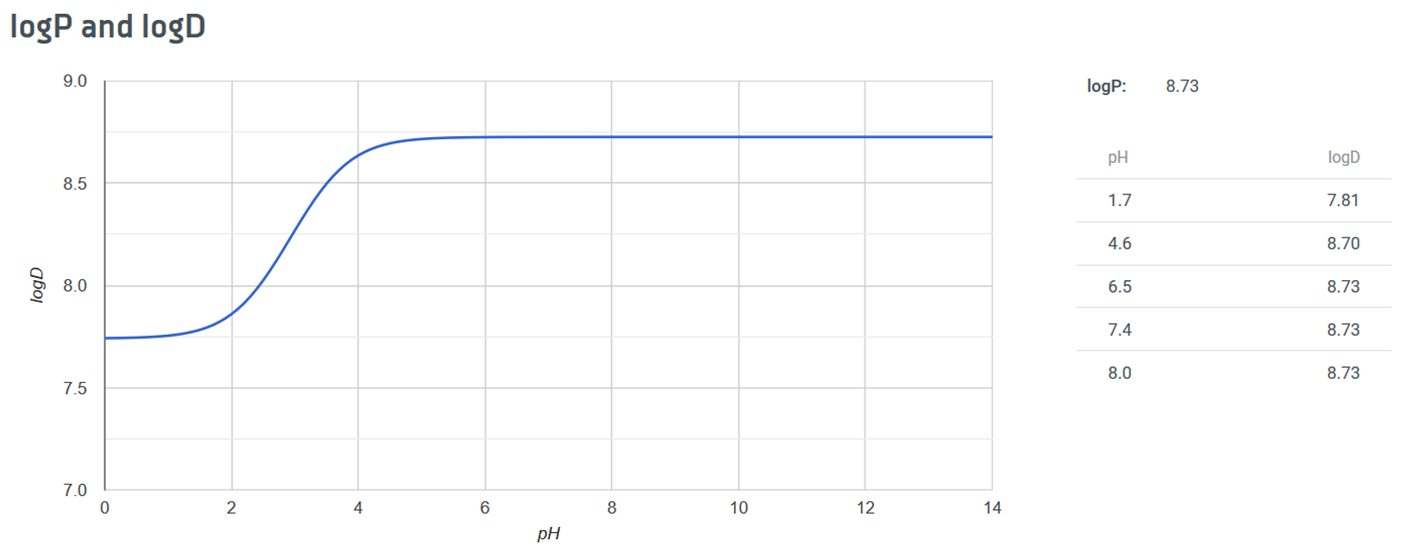


Lupeol succinate (5) ^1^H NMR (CDCl_3_) δ [ppm]: δ9.67(s, 1H, H-37) δ4.70(t, 1H, H-6) δ4.54(m, 2H, H-5) δ2.68(m, 2H, H-35) δ2.61(m, 2H, H-34) δ2.36(m, 1H, H-2) δ1.87(m, 1H, H-9) δ1.68(s, 3H, H-23) δ1.65(m, 6H, H-27,28) δ1.58(m, 2H, H-3) δ1.49(m, 1H, H-11) δ1.45(m, 1H, H-17) 1.40(d, 4H, H-10,14) δ1.31(dd, 4H, H-8,13), 1.21(m, 4H, H-16,18) δ1.02(s, 3H, H-24) δ0.97(d, 1H, H-21) δ0.94(s, 3H, H-26) δ0.84(m, 8H, H-22,30,31), 0,77(d, 4H, H-19,20). ^13^C NMR (CDCl_3_) δ [ppm]: 177.42(C-36), 171.79(C-32), 150.94(C-25), 109.33(C-30), 81.59(C-6), 55.37(C-21), 50.30(C-31), 48.26(C-4), 47.99(C-5), 42.98(C-1), 42.81(C-7), 40.82(C-12), 39.98(C-15), 38.33(C-17), 37.96(C-11), 37.82(C-9), 37.05(C-2), 35.55(C-3), 34.17(C-20), 29.31(C-35), 29.81(C-19), 28.93(C-10), 27.87(C-13), 27.41(C-14), 25.06(C-8), 23.61(C-16), 20.93(C-18), 19.27(C-27), 18.17(C-28), 17.99(C-22), 16.49(C-34), 16.15(C-26), 15.96(C-23), 14.51(C-24). IR max (cm^-1^): 3072.89, 2939.79, 2865.40, 2853.11, 2632.86, 1731.94, 1638.80, 1606.83, 1451.25, 1381.34, 1391.31, 1365.10, 1317.04, 1277.34, 1244.99, 1197.91, 1148.10, 1104.44, 1078.81, 1068.60, 1025.09, 1012.26, 978.87, 943.35, 900.85, 874.53, 806.34, 745.43, 702.47, 658.98. Calculated for C34H54O4 (526.79): C, 79.55%; H, 9.59%; found: C, 80.94%; H, 9.42%. MS-APCI: m/z = 423.3 [M + H]+ (100%), calc 423.3


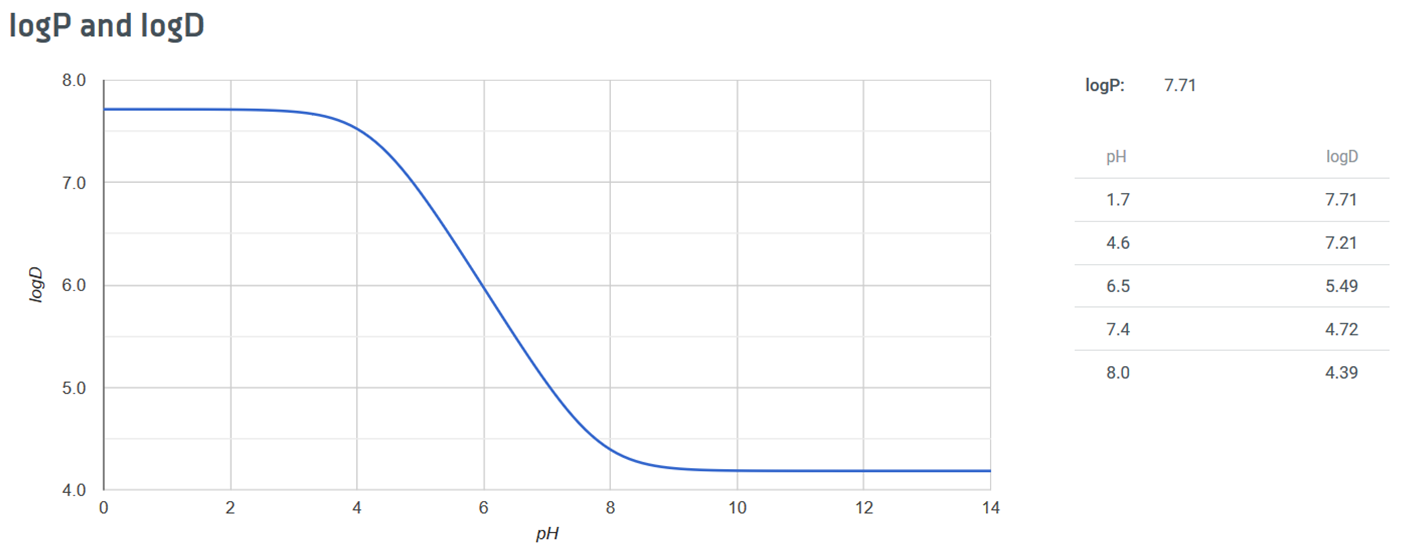


**Lupeol acetylsalicylate (6)** **^1^H NMR** (CDCl_3_) δ [ppm]: δ8.11 (d, 1H, H-36) δ7.63 (t, 1H, H-39) δ7.35 (t, 1H, H-38) δ7.12 (dd, 1H, H-37) 4.62 (dd, 2H, H-5) δ3.21(t, 1H, H-6) δ2.39(t, 2H, H-2) δ2.31(s, 6H H-23,24) δ2.29(s, 3H, H-12) δ2,28(s, 3H, H-31) δ2.08(t, 1H, H-9) δ1.90(q, 2H, H-10) δ1.68(s, 3H, H-27) δ1.60(m, 3H, H-28) δ1.46(t, 2H, H-3) δ1.37(m, 4H, H-8,13) δ1.30(q, 1H, H-11) 1.26 (t, 1H, H-17) δ1.15(dd, 2H, H-14) δ1.03(s, 3H, H-26) δ0.98 (s, 1H, H-30) δ0.95(d, 4H, H-16,18) δ0.84(m, 1H, H-21) δ0.83(s, 3H, H-22) δ0.79(m, 4H, H-19,20). **^13^C NMR** (CDCl_3_) δ [ppm]: 169.53(C-32), 169.03(C-41), 150.88(C-25), 136.67(C-42), 134.85(C-34), 134.46(C-35), 132.51(C-36), 130.87(C-37), 126.14(C-38), 123.98(C-39), 109.30(C-30), 79.16(C-6), 55.26(C-21), 50.41(C-31), 48.28(C-4), 47.97(C-5), 42.98(C-1), 42.75(C-7), 40.81(C-12), 39.99(C-15), 38.81(C-17), 38.63(C-11), 38.03(C-9), 37.14(C-2), 35.56(C-3), 34.25(C-20), 29.80(C-19), 27.96(C-10), 27.43(C-13), 27.24(C-14), 25.12(C-8), 20.98(C-16), 19.29(C-18), 18.29(C-27), 17.99(C-28), 16.10(C-22), 15.96(C-26), 15.36(C-23), 14.53(C-24). **IR max** (cm^-1^)**:** 3359.91, 3068.83, 2977.26, 2934.54, 2891.62, 2867.79, 2846.73, 2671.81, 2598.26, 1725.63, 1708.96, 1640.70, 1483.90, 1465.36, 1444.83, 1412.12, 1381.60, 1365.01, 1354.28, 1252.16, 1314.70, 1264.80, 1233.07, 1211.04, 1174.52, 1146.39, 1105.98, 1067.10, 1039.37, 1022.48, 1006.59, 976.40, 940.14, 923.64, 880.51, 847.77, 806.53, 767.59, 741.29,729.66, 714.00, 672.80. **Calculated for** C39H56O4 (588.86): C, 77.52%; H, 10.33%; found: C, 76.86%; H, 10.03%. **MS-APCI**: m/z = 409.3 [M + H]+ (100%), calc 409.3


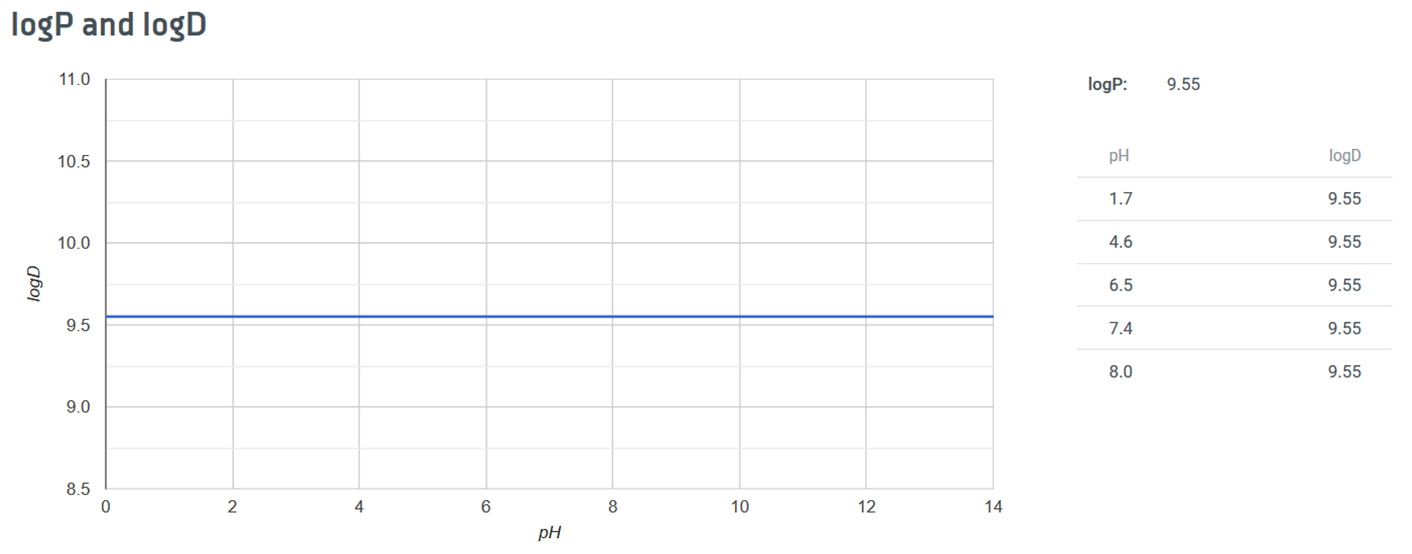


The supplementary crystallographic data for 2, 3 and 5 CCDC 1487997-9. can be obtained free of charge from the Cambridge Crystallographic Data Centre via [www.ccdc.cam.ac.uk/data_request/cif](http://www.ccdc.cam.ac.uk/data_request/cif).
